# A quantum leap in cancer vaccines?

**DOI:** 10.1186/s40425-016-0192-3

**Published:** 2016-12-20

**Authors:** Eli Gilboa

**Affiliations:** University of Miami School of Medicine, Miami, USA

Vaccination, namely the controlled activation of antigen-specific immune cells, is a first and critical step toward engendering protective immunity against cancer. A large number of vaccination strategies developed to date, from simple to complex, have shown but limited efficacy. In a recent article in Nature, Kranz and colleagues describe what appears to be a remarkably effective and relatively simple approach, using tumor antigen encoding mRNA complexed to cationic lipid carriers, RNA-lipoplexes (RNA-LPXs) like DOTMA, DOPE and DOTAP [1]. Vaccination with such mRNA-lipid complexes, exhibiting a net positive charge that was shown to improve mRNA delivery in vitro, have been previously used but were not particularly effective in murine tumor immunotherapy studies. What was different here? Amazingly and counter intuitively, all it took was to tweak the net charge of the RNA to lipid ratio to be slightly negative. This is not to take away from the thorough and extensive study and the foresight of the investigators that led to this discovery. Flies in the face of the common wisdom that therapeutic advances in cancer will require complex and elaborate protocols such as patient-specific ex vivo generated dendritic cells based vaccines.

The main finding was that the intravenously injected negatively charged anionic, but not the conventional neutral or positively charged cationic mRNA lipoplexes were taken up almost exclusively by CD11c + macrophages and dendritic cells (DC) residing in the spleen, lymph nodes and other organs like the liver. This was accompanied by exceptionally strong and durable T cell responses unlike what has been previously seen. The mechanism was dependent on macropinocytosis-mediated uptake of the RNA-encoded antigens by the DC. Macropinocytosis, which is constitutively active in immature DC and downregulated upon their maturation, is unique in its ability to efficiently promote the translocation of its cargo to the cytoplasm, and plays a key role in cross-presentation of exogenous class II, and probably class I, antigens. Uptate of the RNA-LPXs by the DC led to a TLR7 dependent induction of IFNα presumably triggered by the encapsulated mRNA, which led to their maturation and ability to optimally activate T cells. Local and transient expression of IFNα is a key mediator promoting the induction of potent T cell immunity against blood-borne pathogens [2] and tumors [3]. At about the same time, Broos and colleagues have published a study that parallels the findings in this paper using a commercially available lipid formulation, Lipofectamine RNAiMAX, showing that the encapsulated mRNA in the negatively charged RNA-lipoplexes is targeted to phagocytic cells in the spleen and liver, translated mainly in CD11c^+^ DC, induces IFNα, and stimulates a T cell response [4].

This rather simple and cost-effective vaccination protocol using readily available reagents offers a number of important advantages: (i) It dispenses with the need to develop and use a targeting ligands to suitable receptors like DEC205 or Clec9A that are expressed on the surface of resident DC [5]. (ii) Delivering antigens to DC in situ by conjugation of the antigen to targeting ligands can stimulate potent immunity but requires the use of adjuvants to activate the targeted DC such as anti-CD40 antibodies administered systemically [5]. The RNA-LPX provides its own adjuvant, the mRNA itself, to locally and transiently induces IFNα. (iii) Direct injection of antigen encoding mRNA, not DNA, is a surprisingly effective and straightforward approach to induce (tumor) immunity in vivo [6]. (iv). Encapsulation of the mRNA into the lipoplexes protects the unmodified mRNA from nuclease degradation. (v). Lastly, the nanoparticle size RNA-LPXs are efficiently and preferentially taken up by phagocytic cells including dendritic cells.

Ultimately the question is whether the RNA-LPX vaccine will elicit clinical responses in cancer patients that are superior to what we have seen so far. In transplantable murine tumor models vaccination in prophylactic and therapeutic settings suggests that the approach has merit, but the conditions were not particularly stringent so the potency was difficult to asses. More impressive were early results from an ongoing clinical trial in melanoma patients. Three patients treated with low to moderate doses of the RNA-LPX vaccine targeting 4 melanoma antigens exhibited robust and durable T cell responses, in one patients reaching levels comparable to that of CMV and EBV T cell responses that are thought to be protective. Such responses appear to be more robust than what has been generally seen in similar settings but it is hard to judge. Evaluation of more patients, treated with higher doses of vaccine, and signs of clinical responses, are eagerly awaited.

It is prudent to assume that the RNA-LPX vaccination as a stand alone therapy will not be the sought for “magic bullet”, but rather an important component in a multi-pronged approach. One challenge is to improve the vaccine induced immune response, expanding the vaccine activated T cells thru 4-1BB, OX40, GITR or CD27 costimulation, and extending its persistence by skewing the differentiation of the vaccine-activated T cells to become long lasting memory cells [7], especially (tumor) resident memory T cells [8]. Not less and probably more important will be to enhance the susceptibility of the patients’ disseminated tumor lesions to the vaccine generated T cells, countering immune resistance (not limited to checkpoint blockade), and overcoming tumor induced barriers that prevent T cells and antigen presenting cells from penetrating into the tumor mass [9].

Initial evidence from the murine studies and the ongoing clinical trial is that the RNA-LPX vaccine will be safe. There may be some risk that the systemically administered RNA-LPX could elicit excessively high levels of IFNα by the CD11c + cells scattered throughout the body that could provoke autoimmune sequalae especially in predisposed individuals. This can be minimized by targeting the RNA lipoplexes to the tumor lesions using suitable ligands decorating the lipoplexes that bind to tumor secreted products such as VEGF or osteopontin [10]. Tumor targeting may be worth considering also in combination with systemic delivery to enhance the immunogenicity of tumor lesions. This is based on studies showing that intratumoral administration of STING ligands can elicit tumor immunity via local induction of IFNα [11]. Targeting the RNA lipoplexes to tumor lesions, in addition to systemic delivery, could serve a similar purpose.
